# From GWAS Signals to Molecular Mechanisms: Explainable AI for Causal Gene Prioritization and Biomolecular Target Interpretation

**DOI:** 10.3390/biom16071029

**Published:** 2026-07-14

**Authors:** Mia Yang Ang, Li Chen, Lanni Song, Leonard Lipovich, Siew Woh Choo

**Affiliations:** 1 Department of Biomedical Sciences, Jeffrey Cheah Sunway Medical School, Faculty of Medical and Life Sciences, Sunway University, Sunway City, Petaling Jaya 47500, Selangor, Malaysia; miayanga@sunway.edu.my; 2 Sunway Microbiome Centre, Faculty of Medical and Life Sciences, Sunway University, Sunway City, Petaling Jaya 47500, Selangor, Malaysia; 3 Zhejiang-Malaysia Joint Laboratory for Rare Medicinal Resources, Wenzhou-Kean University, 88 Daxue Road, Ouhai, Wenzhou 325060, China; 4 College of Science, Mathematics and Technology, Wenzhou-Kean University, 88 Daxue Road, Ouhai, Wenzhou 325060, China; 5 Dorothy and George Hennings College of Science, Mathematics and Technology, Kean University, 1000 Morris Ave, Union, NJ 07083, USA; 6 International Frontier Interdisciplinary Research Institute (IFIRI), Wenzhou-Kean University, 88 Daxue Road, Ouhai, Wenzhou 325060, China

**Keywords:** GWAS, SNP, complex traits, eQTL, explainable artificial intelligence, causal gene prioritization, molecular genetics, regulatory genomics, drug target prioritization, biomarker discovery

## Abstract

Genome-wide association studies (GWAS) have identified thousands of loci associated with complex human diseases. However, the majority of the association signals reside in non-coding regions of the genome, and do not directly reveal the causal variant, effector gene, regulatory biomolecule, cell type, pathway, biomarker, or therapeutic target. Because many disease-associated variants act through non-coding regulatory mechanisms, post-GWAS interpretation increasingly depends on fine-mapping, expression quantitative trait loci, transcriptome-wide association studies, and functional evidence from single-cell multi-omics, network biology, and genetic target prioritization. Artificial intelligence can attempt to integrate these heterogeneous molecular evidence layers, but the resulting black-box prediction is insufficient when outputs cannot be biologically reproduced or experimentally tested. This review evaluates explainable artificial intelligence (XAI) as a framework for linking genetic association signals to molecular mechanisms and causal gene hypotheses. We argue that explainability is best treated as a biological requirement because useful models must expose evidence paths from significant disease-associated variants to regulatory elements, genes, transcripts, proteins, pathways, cell states, and therapeutic hypotheses. By emphasizing transparent evidence provenance, ancestry-aware interpretation, and functional validation, XAI can support the translation of GWAS signals into molecularly testable hypotheses for target prioritization and precision molecular medicine. The review focuses on the question of how to accomplish AI-accelerated functionalization of GWAS outputs across complex human diseases and traits.

## 1. Introduction

Genome-wide association studies (GWAS) have become central to modern human genetics because they enable the systematic identification of genetic loci associated with complex diseases, quantitative phenotypes, and clinically relevant traits. However, the biological interpretation of GWAS findings remains challenging, particularly because statistical association alone rarely explains the causal variant, effector gene, regulatory mechanism, or disease pathway. This review focuses on the post-GWAS interpretation problem and examines how explainable artificial intelligence (XAI) may support the translation of genetic association signals into mechanism-aware, biologically testable hypotheses for biomarker discovery, target prioritization, and precision molecular medicine.

### 1.1. Genome-Wide Association Studies and the Post-GWAS Interpretation Challenge

Genome-wide association studies (GWAS) compare—using whole-exome arrays, whole-exome sequencing, and, increasingly, whole-genome sequencing—large case and control cohorts to identify specific alleles of genetic variants, typically SNPs (single-nucleotide polymorphisms), that are statistically significantly associated with a defined quantitative phenotype, disease risk, or trait. This powerful method has transformed human genetics by identifying loci associated with complex diseases and quantitative traits, but association discovery alone rarely identifies the causal variant, effector gene, regulatory molecule, or disease mechanism [1]. The central post-GWAS challenge is therefore to convert statistical association signals into molecular hypotheses that can be tested in appropriate biological systems [2].

This challenge is particularly important because gene exons account for a mere 1.5% of the 3.3 Gbp of DNA sequence that comprises the human genome [3], a fact which leads to the realization that the majority of significantly disease-associated genetic variants necessarily reside outside of protein-coding regions [4]. The human gene catalogs have undergone a significant expansion in the past decade: Gencode now includes 80,000 genes, 75% of which are non-coding RNA (ncRNA) genes [5], meaning that some formerly mysterious significant GWAS variants must now be re-interpreted because of their exonic localization within newly catalogued ncRNA genes that therefore emerge as the direct causal candidates. However, since most GWAS variants remain, even against this background of the enhanced human gene catalogs of the post-genomic era, intronic or intergenic, there are two alternative explanations of how their association with the disease or trait being analyzed arose: either the significant genetic variants are in linkage disequilibrium (LD) with other sequence elements that are directly and causally functional with respect to the phenotype or trait being studied, meaning that the GWAS SNPs serve solely as genetic markers, or, if not located in gene exons, they may influence enhancer activity, promoter usage, chromatin accessibility, transcription-factor binding, RNA expression, splicing, protein abundance, or pathway state [6]. The biological unit of interpretation is no longer a single lead index SNP or the nearest gene, but an “evidence field” that may include credible variants, regulatory elements, target genes, molecular QTLs, cell states, and competing mechanistic explanations [7].

### 1.2. From Manual Annotation to Explainable Artificial Intelligence

**Figure 1:**
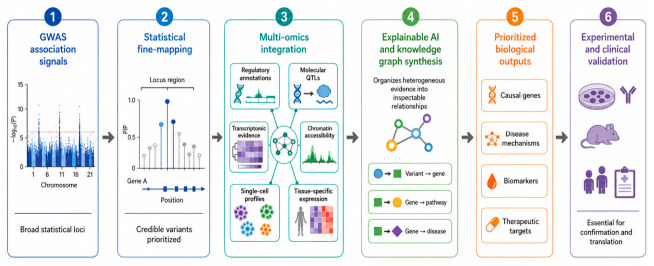
Explainable post-GWAS workflow for translating association signals into molecular hypotheses. GWAS identifies broad statistical loci that can be refined through statistical fine-mapping to prioritize credible variants. Multi-omics integration then links candidate variants to regulatory annotations, molecular QTLs, transcriptomic evidence, chromatin accessibility, single-cell profiles, and tissue-specific expression. Explainable AI models and knowledge graph synthesis organize these heterogeneous evidence layers into inspectable variant-to-gene, gene-to-pathway, and gene-to-disease relationships. This evidence chain supports prioritization of causal genes, disease mechanisms, biomarkers, and therapeutic targets, while emphasizing the need for experimental and clinical validation.

Previously, manual annotation of these multiple evidence layers using the UCSC Genome Browser was used to assign significant disease variants to functional domains of novel genomic features, such as evolutionarily non-conserved non-coding RNA genes, that withstood the test of functional validation in the laboratory and now provide a direct-causality explanation for the underlying GWAS phenotype [8]. Artificial intelligence is expected to be able to help integrate these heterogeneous data layers rapidly and without the observer biases and inefficiencies inherent in manual annotation. Nevertheless, predictive accuracy is insufficient when the model cannot explain why a variant, gene, tissue, pathway, biomarker, or target was prioritized [9]. In post-GWAS interpretation, explainable artificial intelligence (XAI) should expose evidence paths from variants to regulatory elements, genes, transcripts, proteins, pathways, cell states, and validation experiments [10]. This review evaluates XAI as a mechanism-aware framework for translating GWAS signals into molecularly testable hypotheses for biomarker discovery, target prioritization, and precision molecular medicine [11]. The mechanism-aware post-GWAS workflow from initial association discovery to biological prioritization and validation is summarized in Figure 1.

This article is a narrative, mechanism-focused review rather than a formal systematic review or meta-analysis. Relevant research was identified from PubMed, Web of Science, Google Scholar, and major genomics and machine-learning journals using combinations of terms including GWAS, post-GWAS, fine-mapping, colocalization, eQTL, TWAS, causal gene prioritization, explainable artificial intelligence, interpretable machine learning, SHAP, LIME, attention, graph neural network, knowledge graph, DeepSEA, Enformer, Open Targets, CRISPR, and MPRA. We prioritized primary studies, major resources, methodological reviews, and recent examples that directly connect genetic association signals to molecular mechanisms, causal gene prioritization, functional validation, or therapeutic interpretation. Because the aim was conceptual integration across statistical genetics, molecular biology, and XAI, studies were selected for relevance to post-GWAS interpretation rather than pooled quantitatively.

## 2. Molecular Genetics of Complex Traits: From Loci to Mechanistic Interpretation

Complex trait genetics has moved from locus discovery toward molecular interpretation. Early GWAS established that common genetic variation contributes reproducibly to disease risk and quantitative traits, but they also revealed that statistically associated loci often explain only part of disease biology [12]. Increasing sample sizes have expanded discovery across anthropometric, metabolic, cardiovascular, immune, and neurological traits, increasing the number of known significant disease- or trait-associated SNPs and making it possible to fine-map the population-level genetic architecture of these diseases and traits, but biological interpretation—which is essential in order to proceed from significant GWAS SNPs to druggable targets for precision medicine—still depends on connecting association statistics to genes, molecules, tissues, and pathways [13].

The present phase of the field is therefore not simply larger GWAS. It is a shift toward explaining why a locus matters, which biomolecule carries the signal, which cell state is relevant, and how the inferred mechanism could be tested or translated [14]. Early examples of this paradigm included *FTO* [15] and *LOC157273* [8], where SNPs at or near protein-coding genes were functionally demonstrated to control non-coding regulatory elements responsible for other, distant protein-coding genes, and non-coding RNA genes, respectively, in a manner different from that suggested by simplistic annotation of their initial localization. This shift is necessary because exonic SNPs that pinpoint directly causal variants and reveal the disease function of the genes they are inside of comprise the absolute minority of the disease SNPome, whereas the non-coding and often non-transcribed genetic environment of the great majority of other disease-associated SNPs required a nuanced, multidimensional interpretation of all layers of evidence for causality or functionality.

### 2.1. Missing Heritability, Polygenicity, and Regulatory Network Architecture

The missing-heritability problem exposed the gap between inherited risk estimated from population or family data and the fraction explained by significant risk-associated GWAS loci. Part of this gap reflects the basic genetic architecture of common disease (many variants, each with a small effect, are distributed across the genome and additively influence risk), but another part reflects incomplete modeling of regulatory context, gene–gene relationships, ancestry, environment, and molecular intermediates [16].

The omnigenic model sharpened this issue by proposing that many peripheral genes can affect complex traits through regulatory networks that converge on more disease-relevant core genes [17]. Empirical work on trans effects supports the view that gene regulation can distribute genetic influence across broad molecular systems [17]. Empirical work on trans effects supports the view that gene regulation can distribute genetic influence across broad molecular systems [18]. Protein interaction maps and pathway analyses can therefore help distinguish genes that are merely close to a GWAS signal from genes that plausibly participate in the disease-relevant molecular architecture [19].

### 2.2. Mechanism-Aware Precision Medicine and Molecular Trait Interpretation

Precision medicine requires more than inherited-risk prediction. Genetic findings translate into therapeutics when they clarify disease mechanism, biomarker biology, drug response, or patient subgroup structure [20]. Polygenic prediction can stratify risk, but the same score may hide distinct routes through lipid metabolism, adipose biology, immune regulation, vascular biology, neuronal signaling, or pancreatic beta-cell function [21].

This limitation is especially important across populations because score performance, calibration, and biological interpretation can vary when discovery cohorts do not represent the target population [22]. Mechanism-aware precision medicine, the key for progressing from GWAS to new drug targets, therefore requires interpretable evidence layers that connect variants to regulatory annotations, genes, molecular phenotypes, disease tissues, and validation evidence [23].

## 3. Fine-Mapping the Molecular Basis of GWAS Signals

Fine-mapping is a central post-GWAS strategy because association peaks often contain many variants in linkage disequilibrium. Its purpose is to move from a broad locus to a smaller set of statistically plausible causal variants while preserving uncertainty, in contrast to the prior status quo of merely treating the sentinel variant as residing within the putative causal element [24]. This statistical narrowing is necessary, but it is not the same as a molecular explanation [25].

A credible set may prioritize variants without identifying regulatory activity, target gene identity, cell-type relevance, direction of effect, or pathway context. The GWAS-to-function gap persists because statistical resolution and biological resolution are different tasks [2]. XAI can help only when it states whether it is explaining a statistical candidate, a molecular mediator, or a mechanism supported by independent functional evidence [26].

### 3.1. Bayesian Fine-Mapping and Credible Causal Variant Sets

Bayesian fine-mapping assigns posterior probabilities to candidate variants and summarizes uncertainty using credible sets. This approach is preferable to reporting a single sentinel SNP because it shows whether a locus has one dominant candidate or several variants that may reside within the same candidate functional feature and remain difficult to disentangle statistically [27]. Credible sets also create an interface for XAI because model explanations can be compared with posterior genetic evidence [28].

However, credible sets can remain large in regions of extended linkage disequilibrium or limited ancestral diversity. Functional annotations, chromatin accessibility, splicing effects, and disease-relevant molecular QTLs can help rank candidates, but these layers must be reported as evidence rather than proof [29]. Functional genomics and fine-mapping become most useful when they converge on the same regulatory variant, target gene, and cell context [30].

### 3.2. Multi-Ancestry Fine-Mapping and Molecular Interpretation

Multi-ancestry fine-mapping is essential because linkage disequilibrium patterns, allele frequencies, and tagging relationships differ across populations. Diverse cohorts can improve localization of shared signals and reveal ancestry-specific signals that would be missed in a single-population design [31]. Lipid genetics illustrates how broader ancestry representation improves both discovery and resolution [32]. Multi-ancestry stroke analyses also show how broader sampling can identify shared and subtype-specific loci that are more informative for downstream biology [33]. Trans-ancestry fine-mapping paired with molecular assays can further test whether candidate regulatory variants have functional effects consistent with the statistical signal [34].

Ancestry also affects molecular interpretation. Trans-ethnic analyses of blood-cell traits show that biological pathways may be shared even when tagging variants and effect sizes differ across populations [35]. Admixture and local ancestry can affect eQTL discovery and colocalization, so XAI explanations need to report ancestry context for GWAS cohorts, QTL panels, reference LD, and validation samples [36].

### 3.3. Polygenicity, Infinitesimal Effects, and Molecular Architecture

The genetic architecture of complex traits is typically given by many variants, each of which only carries a small effect, which exert their phenotype associations through additive effects and interactions; this complicates the causal interpretation of any given significant variant. Methods that focus only on genome-wide significant loci may miss diffuse regulatory contributions that shape disease biology through weak but widespread molecular perturbations [37]. This does not make mechanism irrelevant; rather, it makes evidence integration more important as a requirement for identifying the mechanism [38].

XAI must therefore clarify whether a prediction depends on a few high-confidence molecular links, a distributed pathway pattern, or a broad polygenic background with limited mechanistic specificity. Functional annotation can improve interpretability when it identifies the regulatory or pathway features that carry risk rather than merely increasing predictive accuracy [39]. The main post-GWAS approaches used to move from statistical association toward molecular interpretation are summarized in Table 1.

## 4. Regulatory Biomolecules Linking Variants to Genes

Regulatory interpretation is the bridge between statistical genetics and molecular mechanism. Because most disease-associated variants are non-coding, the fundamental challenge in relating GWAS to function is to identify how a DNA change alters a regulatory element, transcript, protein, pathway, or cell state in a disease-relevant context [56]. This is difficult because regulatory variants can act at long distances, in specific tissues, during particular developmental windows, or only under stimulation [57]. Chromatin conformation studies show that disease-associated variants can physically connect to distal target genes in primary human cells, replacing simple genomic proximity with regulatory contact evidence [58]. Enhancer maps have also linked risk variants to disease genes across many traits, but these maps should be treated as evidence layers rather than final proof of causality [7].

### 4.1. Molecular QTLs as Intermediates Between Genetic Variation and Biomolecular Function

Molecular QTL mapping connects inherited variants to measurable molecular phenotypes. Expression QTLs, splicing QTLs, chromatin accessibility QTLs, methylation QTLs, protein QTLs, and metabolite QTLs can all help explain how genetic variation changes biological function [45]. Large-scale cis and trans eQTL analyses show that regulatory effects are widespread across tissues and immune contexts [46].

Modern QTL catalogues make these data more systematic by harmonizing datasets across tissues, cell types, and molecular assays [47]. However, QTL evidence is not automatically causal evidence because co-regulation among neighboring genes can obscure the true disease mediator [59]. Single-cell QTL resources add resolution by mapping regulatory effects in cell populations that may be diluted in bulk tissue [60].

### 4.2. TWAS, Colocalization, and Shared Molecular Signal Interpretation

TWAS estimates genetically regulated gene expression and tests whether predicted expression is associated with a trait, providing a gene-level bridge from GWAS to transcriptomic biology [61]. TWAS results must be interpreted cautiously because associations can arise from linkage disequilibrium, shared predictors, or multiple regulatory signals rather than direct transcriptional mediation [44].

Colocalization tests whether a GWAS signal and a molecular QTL signal are consistent with a shared causal variant [62]. Loci with multiple independent signals can weaken simple colocalization assumptions, so methods that model multiple signals are often needed [63]. Recent reviews emphasize that TWAS, colocalization, and related summary-data methods answer related but distinct questions [64]. Methodological overviews of TWAS and post-GWAS gene prioritization reinforce the need to interpret transcript-level associations alongside colocalization and functional data [65]. Recent TWAS reviews also emphasize tissue relevance, LD-mediated confounding, and cross-tissue interpretation as recurring constraints [66].

### 4.3. Cell-Type and Spatial Resolution of Regulatory Mechanisms

Bulk tissue analyses can obscure the cell type or cell state in which a genetic signal operates. Single-cell epigenomic and transcriptomic approaches improve post-GWAS interpretation by locating regulatory annotations, expression effects, and disease enrichment within specific cell populations [67]. Single-cell chromatin accessibility atlases provide feature spaces for explaining variant effects through accessible chromatin, motif disruption, and cell-type enrichment [68]. Disease-specific single-cell epigenomic studies illustrate how genetic risk can be interpreted through immune cell states and regulatory programs [69].

Chromatin architecture and spatial transcriptomics add further context. Regulatory elements may act through three-dimensional contacts rather than linear proximity [70]. Spatial transcriptomics preserves tissue architecture, which can matter when disease-relevant cell states are organized around vascular niches, immune infiltrates, fibrotic regions, or organ-specific microenvironments [71]. Broader spatial transcriptomics reviews show that tissue architecture can change how cellular expression states are interpreted in complex disease systems [54].

## 5. Molecular Networks, Polygenic Risk, and Knowledge Graphs

Polygenic risk scores, network biology, and knowledge graphs organize distributed genetic evidence in different ways. A PRS summarizes many variants into a risk prediction, a network maps relationships among molecules and pathways, and a knowledge graph links variants, genes, diseases, drugs, tissues, and evidence sources [20]. These approaches become more useful when connected rather than treated separately [72].

For molecular interpretation, the key question is not only whether a score predicts disease. The key question is whether inherited risk can be decomposed into regulatory DNA, RNA expression, protein abundance, metabolic pathways, immune activation, vascular biology, or another mechanism [73].

### 5.1. Polygenic Risk Scores and the Limits of Molecular Interpretability

PRS can stratify inherited susceptibility, but clinical prediction and mechanistic explanation are not the same. A high score can identify risk enrichment while leaving unclear whether risk is driven by adipose distribution, insulin secretion, lipid metabolism, inflammation, vascular biology, or another route [21]. Pathway-specific scores can improve interpretability by decomposing aggregate liability into biological components [74].

Portability remains a major limitation. Scores trained largely in one ancestry group can perform less well in other groups and may also yield misleading biological interpretations if locus effects or tagging patterns differ. Recent guidance emphasizes fairness, transparency, and reproducibility in polygenic score development and reporting [23].

### 5.2. Network and Pathway Architectures for Biomolecular Mechanism Prioritization

Network biology provides a mechanism-oriented way to interpret polygenic risk because genes and proteins act in connected systems. Instead of asking only which gene is closest to a variant, network analysis asks whether associated genes converge on pathways, protein complexes, regulatory modules, or disease-relevant cellular processes [72]. This perspective is compatible with omnigenic architecture because peripheral genes may influence traits through network paths that converge on core processes [19].

Functional annotations can reveal shared regulatory contexts across diseases and help explain pleiotropic effects [75]. In cardiometabolic genetics, pleiotropy and sex-specific effects at lipid loci illustrate why pathway architecture is often more informative than single-gene interpretation [32].

### 5.3. Knowledge Graphs for Transparent Molecular Evidence Provenance

Knowledge graphs are useful because they store relationships among variants, genes, regulatory elements, molecular phenotypes, drugs, diseases, tissues, and publications. Their value lies not only in prediction but also in evidence provenance [73]. Open target-prioritization resources show how genetics, molecular data, pathways, drugs, safety evidence, and disease annotations can be combined while keeping evidence sources inspectable [14].

Graph-based approaches can reveal connections that are difficult to see in a table of loci, but they are scientifically useful only when curated evidence is distinguished from inferred edges [76]. Graph-based drug-repurposing studies illustrate how harmonized multi-source evidence can support prediction while still requiring transparent source tracing [76]. In target prioritization, transparent evidence provenance helps clarify whether a gene is supported by fine-mapping, QTL colocalization, enhancer contact, pathway membership, druggability, disease expression, or functional validation [77].

## 6. Explainable AI for Biomolecular Mechanism Attribution

Explainable AI is most valuable in post-GWAS biology when it turns model output into a testable biological explanation. A ranked list of genes is insufficient unless the model shows which variants, regulatory elements, molecular QTLs, cell types, pathways, and evidence sources led to each hypothesis [10]. This requirement differs from generic model transparency because molecular genetics requires inspection, reproducibility, and experimental design [11].

Deep learning has shown value in sequence modeling, regulatory prediction, image analysis, and multi-omics integration, but high predictive performance can still reflect correlations that are biologically incomplete or context dependent [9]. XAI becomes useful when it links attribution to biological entities such as motifs, enhancers, genes, cell states, proteins, and pathways [78]. Proteomics and biomarker discovery add an additional interpretation layer because disease mechanisms and drug targets often operate through protein abundance or protein function rather than RNA alone [79].

### 6.1. Operationalizing Explainability in Molecular Post-GWAS Models

In post-GWAS bioinformatics, explainability can be defined as the ability to trace a prediction from genetic association to molecular evidence and biological hypothesis. This includes feature contributions, data sources, assumptions, uncertainty, and validation status behind a prioritized variant, gene, tissue, pathway, biomarker, or target [10].

This definition is stricter than displaying an attention score, saliency map, SHAP value, or graph weight. A visualization is useful only if it can be connected to a biological entity such as a motif, enhancer, transcript, protein, pathway, or cell state [11]. Graph neural networks may be useful for causal gene prioritization, but their explanations must distinguish biological signal from graph topology, training bias, and database curation effects [78]. The major XAI approaches relevant to post-GWAS molecular prioritization differ in their explanation units, biological use cases, and interpretive risks, as summarized in Table 2.

A critical distinction among XAI methods is the unit of explanation and the failure mode that follows from it. SHAP provides additive feature attributions and is useful for ranking variants, genes, annotations, or tissues, but in genomic data it can distribute importance across correlated features in LD or across co-linear functional annotations, making conditional and LD-aware sensitivity analyses essential. LIME can expose local decision rules around a single locus or gene, but its perturbations may generate biologically implausible genotypes or annotation profiles unless constrained by LD, allele frequency, and tissue context. Attention weights can help identify sequence positions, regulatory windows, or graph neighborhoods used by deep models, but attention is not automatically causal and should be compared with gradient, perturbation, or in silico mutagenesis analyses. Graph-based explanations are well matched to gene-regulatory networks, protein interactions, and knowledge graphs because they can identify influential edges, paths, and neighborhoods; however, they are vulnerable to database bias, hub-gene inflation, and evidence leakage from curated resources. Counterfactual explanations are particularly attractive for post-GWAS interpretation because they ask what minimal change in variant, annotation, tissue, or network context would alter a model prediction, but their biological value depends on whether the counterfactual is experimentally plausible and consistent with population genetics.

### 6.2. Biological Accountability, Reproducibility, and Validation of XAI Explanations

XAI can create false certainty if explanations are treated as biological proof. An attribution score may highlight a sequence position, gene, or pathway, but the result remains a model-derived hypothesis until supported by independent molecular evidence [88]. Evidence leakage is another risk because models trained on databases containing GWAS-derived annotations may reproduce prior curation rather than discover new biology [89]. These concerns are consistent with broader recommendations for responsible AI use in life-science bioinformatics, where model-assisted outputs should be evaluated through evidence provenance, reproducibility, expert oversight, risk-aware interpretation, and validation against trusted biological resources [90].

Ancestry and tissue mismatch also matter. Explanations based on reference panels from one ancestry, tissue, or cell state may not transfer cleanly to another population or disease context [36]. Strict biological accountability therefore requires validation outside the model, including biomarkers, perturbation experiments, molecular phenotypes, therapeutic genetics, or clinical pharmacology [91].

For practical use, validation should be matched to the explanation unit. Variant- or enhancer-level attributions should be tested with MPRA, reporter assays, allele-specific chromatin accessibility, or base-editing/prime-editing perturbations when feasible. Transcription-factor or motif-level explanations are best supported by ChIP-seq, CUT&RUN, motif-disruption assays, electrophoretic mobility shift assays, or perturbation of the relevant factor followed by expression profiling. Gene-level explanations should be evaluated with CRISPR knockout, CRISPR interference/activation, RNA interference, overexpression, or perturb-seq in disease-relevant cells. Cell-type or tissue-level explanations require validation in matched cell states using single-cell RNA-seq, single-cell ATAC-seq, spatial transcriptomics, or organoid/model-system assays. Graph or pathway-level explanations should be tested by perturbing key nodes or edges and assessing whether the predicted downstream molecular program changes in the expected direction.

## 7. From Causal Gene Prioritization to Molecular Target Biology

Drug target prioritization is one of the clearest translational uses of post-GWAS interpretation. Human genetic evidence can increase confidence in therapeutic targets because naturally occurring variation links genes, molecular traits, and disease outcomes in human populations [92]. However, a causal gene hypothesis must be connected to direction of effect, tissue of action, disease stage, safety, druggability, and therapeutic modality before it becomes a target hypothesis [91].

XAI can support target prioritization by making clear whether a target was prioritized because of fine-mapping, QTL colocalization, pQTL evidence, pathway membership, disease expression, druggability, or genetic support for efficacy and safety [77]. Target prioritization therefore extends gene prioritization rather than replacing it [89].

### 7.1. Gene Prioritization Versus Therapeutic Target Prioritization

Gene prioritization asks which gene at or near a GWAS locus is most likely to mediate the association. Target prioritization asks whether that gene product can be safely and effectively modulated to change disease biology [73]. A gene may be a credible effector of a locus but still be a poor therapeutic target because its function is pleiotropic, inaccessible, essential in many tissues, or linked to safety liabilities [92].

Open target-prioritization frameworks combine genetics, functional genomics, disease biology, pathway evidence, safety, and druggability while keeping evidence sources inspectable [14]. The most convincing target hypotheses combine genetic association, molecular mediation, tissue relevance, direction of effect, and independent validation [91].

Current post-GWAS interpretation increasingly relies on platforms and models that organize heterogeneous evidence into inspectable prioritization workflows. Open Targets [73] provides a useful example of target-centered evidence integration because it combines genetic association, functional genomics, disease biology, pathway information, tractability, safety, and druggability while retaining evidence provenance. Sequence-based deep learning frameworks such as DeepSEA [93] and Enformer [94] address a complementary problem: they predict regulatory consequences from DNA sequence and can help prioritize non-coding variants by estimating effects on chromatin features, transcription-factor binding, or gene expression. However, these frameworks should not be treated as substitutes for biological validation. Their outputs are most useful when linked back to fine-mapping, QTL colocalization, tissue-specific regulatory maps, and experimental assays that test the predicted variant, enhancer, gene, or pathway.

### 7.2. Mendelian Randomization, Proteomics, and Direction of Molecular Effect

Mendelian randomization uses genetic variants as instruments to estimate whether a modifiable exposure may influence disease risk. In drug development, MR can support or challenge target hypotheses before expensive experimental or clinical studies [95]. Proteomic integration is especially useful because many therapeutic targets are proteins rather than transcripts [96].

Protein-level evidence can connect inherited variation to circulating or tissue-relevant protein abundance and disease outcomes [97]. Similar MR and proteomic strategies have been used to nominate targets in calcific aortic valve disease and atrial fibrillation, illustrating the value of protein-centered target evidence [98]. Genetic target evidence in atrial fibrillation also shows why sensitivity analyses and tissue context remain essential for interpretation [99]. Recent studies in cancer and autoimmune disease illustrate how genetics, proteomics, MR, and colocalization can nominate protein biomarkers and candidate drug targets while still requiring experimental validation [100]. The IL6R example remains a useful benchmark because human genetic evidence anticipated therapeutic modulation of an inflammatory pathway [101]. Multi-omics MR and colocalization studies in autoimmune disease show how target nomination becomes stronger when genetic and molecular evidence converge [102].

### 7.3. Functional Validation of Variants, Genes, Proteins, and Pathways

Functional validation is the step that converts an interpretable prediction into biological evidence. A model can nominate a variant, enhancer, gene, or pathway, but experimental systems are needed to test whether perturbing that molecule changes expression, protein abundance, cell state, or disease-relevant phenotype [103]. CRISPR screens can test gene function at scale and rank mechanisms experimentally [104].

Massively parallel reporter assays are especially useful for non-coding variants because they test allele-specific regulatory activity directly [105]. Modern MPRA designs can evaluate many candidate regulatory elements and variants, providing an empirical check on model explanations [106]. Locus-specific perturbation studies show how a GWAS signal can be connected to a candidate gene and tested experimentally [107]. MPRA studies across psychiatric disorders illustrate how shared non-coding variants can be tested for allele-specific regulatory activity across disease contexts [108]. The major translational evidence layers used to connect post-GWAS gene prioritization with drug target prioritization are summarized in Table 3.

A useful validation workflow is therefore sequential rather than purely computational: first define a credible set through fine-mapping, then connect variants to genes through colocalization, chromatin contact, enhancer activity, and cell-type specificity, then use XAI to nominate the explanatory feature, and finally test the nominated unit experimentally. For a non-coding variant, this may involve MPRA or reporter assays followed by CRISPR editing of the endogenous enhancer and measurement of target-gene expression. For a gene-level explanation, CRISPRi/CRISPRa or perturb-seq can test whether changing the predicted gene alters the disease-relevant cellular phenotype. For a pathway-level explanation, perturbing multiple nodes provides stronger evidence than validating a single highly connected gene.

## 8. Worked Example: Obesity and Cardiometabolic Molecular Mechanisms

Obesity and cardiometabolic disease provide a useful worked example because they combine polygenic architecture, tissue specificity, endocrine biology, metabolic pathways, behavioral regulation, and therapeutic relevance [120]. GWAS has identified many loci for body mass index, adiposity, fat distribution, type 2 diabetes, lipids, and cardiovascular disease, but these signals must be interpreted through molecular mechanisms [13].

Body fat distribution is especially informative because genetic associations point to adipose biology, insulin resistance, vascular risk, and sex-specific effects [121]. Earlier GWAS of body fat distribution linked adipose and insulin biology to differences in cardiometabolic risk [122]. Lipid genetics also shows how pleiotropy and sexual dimorphism can complicate the interpretation of cardiometabolic mechanisms [32]. Type 2 diabetes genetics adds beta-cell function, insulin sensitivity, liver metabolism, adipose biology, and vascular outcomes to the interpretive problem [123]. Cross-ancestry obesity analyses continue to identify genes and pathways that can refine biological interpretation across populations [124]. Integrative prioritization frameworks combining GWAS signals with functional and biological annotation have also been used to identify plausible causal genes and therapeutic targets for obesity [125].

### 8.1. Tissue, Cell-Type, and Organ-Specific Molecular Mechanisms

Cardiometabolic traits involve adipose tissue, liver, pancreas, skeletal muscle, brain, vasculature, kidney, and immune cells. Organ-specific regulatory effects can illuminate cardiometabolic mechanisms when the tissue matches the expected disease biology [126]. Integrating fine-mapping with multi-omics data can reveal candidate effector genes and pathways, but each molecular layer must be interpreted in context [127].

Single-cell transcriptome-wide MR and colocalization approaches extend this logic by linking genetic evidence to cell-type-specific molecular programs [128]. Coronary artery disease genetics shows how loci can be characterized across regulatory annotations, genes, pathways, and vascular biology [129]. Shared genetic susceptibility between abdominal aortic aneurysm and cardiometabolic traits further illustrates how lipid metabolism and inflammation can connect vascular and metabolic disease mechanisms [130]. Integrative functional genomics and fine-mapping of multivariate obesity GWAS can identify regulatory mechanisms with cardiometabolic implications [131].

### 8.2. Explainable AI for Cardiometabolic Mechanism and Target Prioritization

Cardiometabolic genetics is well suited to XAI because the evidence base is large but mechanistically fragmented. Models need to integrate GWAS, fine-mapping, molecular QTLs, metabolomics, proteomics, tissue annotations, pathway networks, targets, and phenotypes while keeping each inference inspectable [132]. Genetic subtyping of obesity shows how similar body mass index values can arise from different biological routes [133].

Multivariate insulin resistance studies show that correlated cardiometabolic traits can reveal loci and pathways that are not obvious from one phenotype alone [134]. AI-driven multi-omics models illustrate both the promise and risk of high-dimensional integration, making explanation quality essential [135]. Multi-omics studies of shared genetic architecture across immune-mediated diseases further show how model explanations can track pleiotropic molecular mechanisms across related conditions [136]. Polygenic prediction models that incorporate functional annotations also show how prediction can become more interpretable when annotations are biologically meaningful [39]. Obesity and cardiometabolic disease provide practical examples of how phenotype-level GWAS signals can be connected to tissue context, molecular evidence, mechanistic interpretation, and translational cautions, as summarized in Table 4.

Obesity and cardiometabolic loci also illustrate how evidence leakage can occur. If a model is trained on databases that already encode GWAS-derived gene-disease links, then high scores for genes such as *FTO* [137], *MC4R* [138], *SORT1* [139], *PCSK9* [140], or *APOE* [141] may partly reflect prior curation rather than independent discovery. Practical mitigation strategies include holding out entire loci, diseases, or evidence sources from training; evaluating predictions in external cohorts not used to build the knowledge base; repeating analyses after removing GWAS-derived annotations; and testing whether synthetic perturbations of non-genetic evidence change the explanation in biologically sensible ways [142]. In a cardiometabolic pipeline, for example, a *SORT1* explanation should remain supported by liver eQTL colocalization, chromatin evidence, and perturbation data even when curated lipid-disease database edges are masked. In an obesity pipeline, candidate genes near *FTO* or *MC4R* should be evaluated in ancestry-stratified and tissue-specific analyses rather than accepted because they are already prominent in the training database.

**Table 4:** Obesity and cardiometabolic examples in a GWAS-to-function pipeline.

Phenotype	Biological Focus	GWAS-To-Function Use	Key Caution	References
Obesity/BMI	Central appetite regulation, energy balance, adipose biology, endocrine signalling	Uses GWAS loci to prioritize neuronal, adipose, endocrine, inflammatory, or metabolic mechanisms underlying body-weight regulation	BMI-associated loci should not be interpreted as a single obesity mechanism or as direct evidence for a therapeutic target	[120]
Body fat distribution	Adipose distribution, adipogenesis, insulin sensitivity, sex-dimorphic fat storage	Interprets waist-to-hip ratio loci in relation to adipose biology, sex-specific regulation, and metabolic risk beyond general adiposity	Fat distribution is not interchangeable with BMI; ancestry, sex, and measurement context can alter interpretation	[121]
Type 2 diabetes	Beta-cell function, insulin secretion, insulin resistance, liver and lipid metabolism	Uses genetic clustering and molecular annotation to distinguish beta-cell, insulin-resistance, adiposity, lipid, and liver-related mechanisms	Genetic clusters may suggest mechanisms but should not be treated as definitive clinical subtypes without validation	[143,144]
Insulin resistance/metabolic syndrome	Hepatic, adipose, skeletal-muscle, lipid, and glucose–insulin regulatory pathways	Integrates multivariate GWAS, QTL evidence, and metabolic traits to separate shared systemic risk from trait-specific signals	Fasting insulin, TyG index, and metabolic syndrome components are imperfect proxies for tissue-specific insulin resistance	[145,146]
Blood lipids/dyslipidaemia	LDL-C, HDL-C, triglyceride metabolism, apoB-containing lipoproteins, hepatic lipid regulation	Uses lipid GWAS, pQTLs, colocalization, and drug-target evidence to prioritize cardiometabolic targets	Direction of effect is essential because increasing or lowering a molecule may have different therapeutic implications	[22,147]
Coronary artery disease	Vascular endothelium, smooth muscle biology, lipid handling, inflammation, atherosclerosis	Links CAD loci to vascular, endothelial, lipid, inflammatory, and network-based mechanisms using epigenomic and perturbation evidence	Prediction models or network rankings should not be interpreted as mechanism without causal and functional support	[148,149]
AI-derived cardiometabolic imaging phenotypes	Retinal vasculature, microvascular morphology, cardiometabolic imaging biomarkers	Uses deep-learning-derived imaging traits to connect vascular image features with cardiometabolic risk and GWAS signals	Image-derived prediction may support biomarker discovery but does not by itself establish disease mechanism or therapeutic causality	[150,151]

## 9. Future Directions

Future post-GWAS interpretation will depend on deeper integration of statistical genetics with molecular biology. The most useful systems will combine fine-mapping, QTL resources, TWAS, colocalization, single-cell data, spatial data, proteomics, metabolomics, networks, knowledge graphs, and perturbation evidence in one inspectable workflow [64]. Reference resources also need to become more diverse and context-aware [47].

XAI methods require stronger biological evaluation standards. Explanation quality should be tested against known regulatory mechanisms, held-out functional assays, perturbation results, and independent disease datasets [10]. Future systems also need uncertainty-aware reporting so that readers can tell whether a gene is strongly supported, weakly supported, contradicted, tissue-specific, ancestry-limited, or awaiting validation [11].

Implementation should move beyond qualitative labels by attaching calibrated uncertainty to both predictions and explanations. Bayesian models can report posterior probabilities for variant-gene, gene-trait, or pathway-trait hypotheses [48,152]; ensemble models can provide confidence intervals or credible intervals for attribution scores; and calibration plots, Brier scores, expected calibration error, and decision-curve analyses can show whether predicted support corresponds to empirical validation rates [153,154,155]. For explanation outputs, investigators should report stability across bootstrap samples, ancestry-stratified reference panels, LD-pruned inputs, tissue contexts, and alternative model classes [156]. These quantities would allow readers to distinguish genes that are strongly supported, weakly supported, contradicted, tissue-specific, ancestry-limited, or awaiting validation.

## 10. Conclusions

GWAS has produced an extensive map of disease-associated loci, but maps alone do not explain biological mechanisms. The central task is to connect association signals to causal variants, regulatory elements, genes, transcripts, proteins, pathways, cell states, biomarkers, and therapeutic hypotheses in a way that can be inspected and tested. Fine-mapping, molecular QTL mapping, TWAS, colocalization, single-cell multi-omics, spatial profiling, network biology, and knowledge graphs each contribute a different part of this evidence chain.

Explainable AI can support this process because it can organize heterogeneous evidence and make the reasoning behind a prediction visible, but explainability is not equivalent to biological truth. Attribution, attention, graph importance, and feature contribution scores must be interpreted as hypotheses unless supported by independent genetic, molecular, and functional evidence.

For this reason, the next generation of post-GWAS XAI studies should report not only the top-ranked genes or variants, but also the validation status, uncertainty, ancestry representation, tissue or cell-state dependence, and potential evidence leakage of each explanation. Functional follow-up such as CRISPR perturbation, MPRA, reporter assays, QTL colocalization in independent cohorts, and cross-ancestry replication should be presented as part of the evidentiary chain that determines whether an AI explanation is biologically credible.

## Figures and Tables

**Table 1:** Major post-GWAS approaches for molecular interpretation.

Method	Primary Input	Mechanistic Contribution	Main Limitation	References
Statistical fine-mapping	GWAS data with LD reference panels	Prioritizes credible sets of candidate causal variants within associated loci	Sensitive to LD structure, ancestry mismatch, sample size, allelic heterogeneity, and model assumptions	[40,41]
Functional annotation and gene-set mapping	Associated variants, gene boundaries, chromatin marks, regulatory annotations, and gene sets	Links non-coding association signals to candidate genes, regulatory elements, pathways, and biological processes	Dependent on annotation completeness, database bias, tissue relevance, and SNP-to-gene mapping assumptions	[42,43]
Transcriptome-wide association study	GWAS summary statistics and tissue-specific expression prediction models	Identifies genes whose genetically predicted expression is associated with disease or traits	May reflect LD contamination, tissue mismatch, correlated expression, or non-causal tagging	[44]
Molecular QTL integration	GWAS data integrated with eQTL, sQTL, pQTL, or mQTL datasets	Connects trait-associated variants to intermediate molecular phenotypes	Limited by tissue specificity, cell-type heterogeneity, QTL panel size, and uncertain directionality	[45,46,47]
Colocalization	GWAS and molecular QTL summary statistics with LD information	Tests whether trait and molecular QTL signals likely share the same causal variant	Sensitive to priors, LD structure, allelic heterogeneity, and single-causal-variant assumptions	[48]
Methylation QTL integration	Genotype, DNA methylation, and trait-association data	Adds an epigenetic regulatory layer by identifying candidate methylation-mediated links	DNA methylation is tissue-specific and affected by environment, cell composition, and reverse causality	[49,50]
Single-cell enhancer–gene mapping	Single-cell or single-nucleus chromatin accessibility and gene-expression data	Resolves candidate enhancer–gene links in specific cell types or cell states	Limited by sparse data, batch effects, incomplete reference maps, and correlation-based inference	[51,52,53]
Perturbation and functional validation	Prioritized variants, genes, enhancers, or regulatory elements tested experimentally	Provides direct experimental evidence for variant, enhancer, gene, or pathway function	Often low-throughput, expensive, context-dependent, and not always performed in disease-relevant systems	[54,55]

**Table 2:** Explainable AI approaches for post-GWAS molecular prioritization.

XAI Technique	Explanation Unit	Post-GWAS Use Case	Main Risk	References
SHAP-based feature attribution	Feature contribution score	Identifies which molecular evidence layers contribute most to variant, gene, or pathway prioritization	Correlated features, LD structure, and redundant omics signals may distort feature importance	[11,78]
LIME/local surrogate explanation	Local explanatory feature subset	Explains why a specific locus, gene, or patient profile was prioritized by a complex model	Explanations may be unstable in sparse, high-dimensional genomic data	[78,80]
Saliency or gradient-based attribution	Nucleotide, motif, sequence window, or regulatory position	Highlights sequence features or regulatory motifs influencing predictions for non-coding variants	Attribution maps may be noisy, model-dependent, or biologically misleading	[81,82]
Attention mechanisms	Attention weight over features, tokens, regions, or biological units	Inspects which genomic regions, cell states, pathways, or molecular features receive higher model weight	Attention weights are not necessarily faithful causal explanations	[78,83]
Graph Attention Networks	Node, edge, or neighborhood contribution	Prioritizes candidate causal genes by integrating GWAS-linked genes with network and omics evidence	Network incompleteness, database bias, and overinterpretation of attention weights may affect inference	[84,85]
Knowledge graph-based explainability	Inspectable evidence path	Traces variant-to-gene, gene-to-pathway, pathway-to-disease, or gene-to-drug relationships	Knowledge graphs may propagate outdated, incomplete, or biased database relationships	[78,86]
Rule-based or surrogate rule extraction	Decision rule or threshold pattern	Converts complex model outputs into interpretable rules for hypothesis generation or risk stratification	Rules may oversimplify nonlinear biology and may not generalize beyond the training dataset	[83,87]
Counterfactual explanations	Minimal feature change needed to alter model output	Tests what molecular evidence would change a gene, locus, or patient prediction from low to high priority	Counterfactuals may appear causal despite being model-dependent perturbations	[80,83]

Note: A practical interpretation of Table 2 is that no single explanation method should be treated as definitive. SHAP and LIME are strongest when the goal is feature-level auditability, attention and sequence perturbation are strongest when the model operates on regulatory sequence, graph explanations are strongest when the hypothesis involves network propagation or pathway structure, and counterfactual approaches are strongest when investigators need experimentally testable alternatives. In post-GWAS settings, the most common quantitative warning signs are attribution instability across LD-pruned or ancestry-stratified inputs, inflated importance assigned to correlated annotations, hub-dominated graph explanations, poor calibration of predicted probabilities, and loss of explanation concordance when the same locus is evaluated across tissues or cell states. Reporting these diagnostics alongside the explanation itself would make the risks in Table 2 more operational for readers and reviewers.

**Table 3:** Evidence layers for post-GWAS drug target prioritization.

Evidence Layer	Translational Contribution	Main Caveat	References
Human genetic disease evidence	Supports early target nomination by linking genes, variants, or pathways to disease risk and mechanism	Gene proximity alone is weak evidence and may misassign the causal gene	[92,109]
Fine-mapping and causal variant prioritization	Refines broad GWAS loci into credible causal variants before assigning candidate target genes	Credible sets may remain broad, especially in regions with strong LD or ancestry mismatch	[40,41]
Colocalization	Tests whether disease and molecular QTL signals share the same causal variant, strengthening target–mechanism inference	Can be misleading with multiple causal variants, weak QTL signals, tissue mismatch, or inappropriate priors	[48]
pQTL and proteomic evidence	Prioritizes protein targets and biomarkers closer to drug action, especially when direction of effect is interpretable	Plasma pQTLs may not reflect tissue-local protein biology, and trans-pQTLs can be difficult to interpret	[110,111]
Drug-target Mendelian randomization	Uses genetic proxies of target perturbation to support target validation, drug repurposing, efficacy prediction, and safety assessment	Weak instruments, horizontal pleiotropy, LD contamination, and lifelong genetic exposure may limit pharmacological interpretation	[112,113]
Single-cell disease-context evidence	Identifies disease-relevant cell types, cell states, and target activity patterns for context-specific prioritization	Single-cell evidence is often sparse, cohort-specific, and correlative	[114,115,116]
Multi-source target–disease evidence integration	Integrates transcriptomic, proteomic, epigenomic, metabolic, and network evidence to build mechanistic target hypotheses	Network proximity is often correlative and can amplify database or literature bias	[73,117]
Druggability and therapeutic tractability	Filters biologically relevant genes into feasible therapeutic targets based on modality, ligandability, and target class	A genetically supported gene may be biologically important but not currently druggable	[118]
Safety genetics and phenome-wide evidence	Anticipates on-target safety liabilities and therapeutic windows across human traits and phenotypes	Genetic proxies may not capture dose, timing, reversibility, or tissue-specific pharmacology	[119]
Functional genomics and perturbation validation	Provides experimental support for mechanism and target actionability through variant, gene, enhancer, or pathway perturbation	In vitro and ex vivo systems may not fully recapitulate human tissue physiology or long-term therapeutic effects	[92,109]

## Data Availability

No new data were created or analyzed in this study. Data sharing is not applicable to this article.

## References

[1] Tam V., Patel N., Turcotte M., Bosse Y., Pare G., Meyre D. (2019). Benefits and limitations of genome-wide association studies. Nat. Rev. Genet..

[2] Broekema R.V., Bakker O.B., Jonkers I.H. (2020). A practical view of fine-mapping and gene prioritization in the post-genome-wide association era. Open Biol..

[3] Gregory T.R. (2005). Synergy between sequence and size in large-scale genomics. Nat. Rev. Genet..

[4] Maurano M.T., Humbert R., Rynes E., Thurman R.E., Haugen E., Wang H., Reynolds A.P., Sandstrom R., Qu H., Brody J. (2012). Systematic localization of common disease-associated variation in regulatory DNA. Science.

[5] Mudge J.M., Carbonell-Sala S., Diekhans M., Martinez J.G., Hunt T., Jungreis I., Loveland J.E., Arnan C., Barnes I., Bennett R. (2025). GENCODE 2025: Reference gene annotation for human and mouse. Nucleic Acids Res..

[6] Zhong W., Liu W., Chen J., Sun Q., Hu M., Li Y. (2022). Understanding the function of regulatory DNA interactions in the interpretation of non-coding GWAS variants. Front. Cell Dev. Biol..

[7] Nasser J., Bergman D.T., Fulco C.P., Guckelberger P., Doughty B.R., Patwardhan T.A., Jones T.R., Nguyen T.H., Ulirsch J.C., Lekschas F. (2021). Genome-wide enhancer maps link risk variants to disease genes. Nature.

[8] Manning A.K., Goustin A.S., Kleinbrink E.L., Thepsuwan P., Cai J., Ju D., Leong A., Udler M.S., Brown J.B., Goodarzi M.O. (2020). A Long Non-coding RNA, LOC157273, Is an Effector Transcript at the Chromosome 8p23.1-PPP1R3B Metabolic Traits and Type 2 Diabetes Risk Locus. Front. Genet..

[9] Ching T., Himmelstein D.S., Beaulieu-Jones B.K., Kalinin A.A., Do B.T., Way G.P., Ferrero E., Agapow P.M., Zietz M., Hoffman M.M. (2018). Opportunities and obstacles for deep learning in biology and medicine. J. R. Soc. Interface.

[10] Novakovsky G., Dexter N., Libbrecht M.W., Wasserman W.W., Mostafavi S. (2023). Obtaining genetics insights from deep learning via explainable artificial intelligence. Nat. Rev. Genet..

[11] Toussaint P.A., Leiser F., Thiebes S., Schlesner M., Brors B., Sunyaev A. (2023). Explainable artificial intelligence for omics data: A systematic mapping study. Brief. Bioinform..

[12] Visscher P.M., Wray N.R., Zhang Q., Sklar P., McCarthy M.I., Brown M.A., Yang J. (2017). 10 Years of GWAS Discovery: Biology, Function, and Translation. Am. J. Hum. Genet..

[13] Yengo L., Sidorenko J., Kemper K.E., Zheng Z., Wood A.R., Weedon M.N., Frayling T.M., Hirschhorn J., Yang J., Visscher P.M. (2018). Meta-analysis of genome-wide association studies for height and body mass index in approximately 700000 individuals of European ancestry. Hum. Mol. Genet..

[14] Mountjoy E., Schmidt E.M., Carmona M., Schwartzentruber J., Peat G., Miranda A., Fumis L., Hayhurst J., Buniello A., Karim M.A. (2021). An open approach to systematically prioritize causal variants and genes at all published human GWAS trait-associated loci. Nat. Genet..

[15] Laber S., Forcisi S., Bentley L., Petzold J., Moritz F., Smirnov K.S., Al Sadat L., Williamson I., Strobel S., Agnew T. (2021). Linking the FTO obesity rs1421085 variant circuitry to cellular, metabolic, and organismal phenotypes in vivo. Sci. Adv..

[16] Wray N.R., Wijmenga C., Sullivan P.F., Yang J., Visscher P.M. (2018). Common Disease Is More Complex Than Implied by the Core Gene Omnigenic Model. Cell.

[17] Boyle E.A., Li Y.I., Pritchard J.K. (2017). An Expanded View of Complex Traits: From Polygenic to Omnigenic. Cell.

[18] Liu X., Li Y.I., Pritchard J.K. (2019). Trans Effects on Gene Expression Can Drive Omnigenic Inheritance. Cell.

[19] Ratnakumar A., Weinhold N., Mar J.C., Riaz N. (2020). Protein-Protein interactions uncover candidate ‘core genes’ within omnigenic disease networks. PLoS Genet..

[20] Torkamani A., Wineinger N.E., Topol E.J. (2018). The personal and clinical utility of polygenic risk scores. Nat. Rev. Genet..

[21] O’Sullivan J.W., Raghavan S., Marquez-Luna C., Luzum J.A., Damrauer S.M., Ashley E.A., O’Donnell C.J., Willer C.J., Natarajan P., Vice Chair on behalf of the American Heart Association Council on Genomic and Precision Medicine (2022). Polygenic Risk Scores for Cardiovascular Disease: A Scientific Statement From the American Heart Association. Circulation.

[22] Duncan L., Shen H., Gelaye B., Meijsen J., Ressler K., Feldman M., Peterson R., Domingue B. (2019). Analysis of polygenic risk score usage and performance in diverse human populations. Nat. Commun..

[23] Xiang R., Kelemen M., Xu Y., Harris L.W., Parkinson H., Inouye M., Lambert S.A. (2024). Recent advances in polygenic scores: Translation, equitability, methods and FAIR tools. Genome Med..

[24] Spain S.L., Barrett J.C. (2015). Strategies for fine-mapping complex traits. Hum. Mol. Genet..

[25] Wang Q.S., Huang H. (2022). Methods for statistical fine-mapping and their applications to auto-immune diseases. Semin. Immunopathol..

[26] Caliskan M., Brown C.D., Maranville J.C. (2021). A catalog of GWAS fine-mapping efforts in autoimmune disease. Am. J. Hum. Genet..

[27] Yang Z., Wang C., Liu L., Khan A., Lee A., Vardarajan B., Mayeux R., Kiryluk K., Ionita-Laza I. (2023). CARMA is a new Bayesian model for fine-mapping in genome-wide association meta-analyses. Nat. Genet..

[28] Stanzick K.J., Li Y., Schlosser P., Gorski M., Wuttke M., Thomas L.F., Rasheed H., Rowan B.X., Graham S.E., Vanderweff B.R. (2021). Discovery and prioritization of variants and genes for kidney function in >1.2 million individuals. Nat. Commun..

[29] Jiang X., Dellepiane N., Pairo-Castineira E., Boutin T., Kumar Y., Bickmore W.A., Vitart V. (2020). Fine-mapping and cell-specific enrichment at corneal resistance factor loci prioritize candidate causal regulatory variants. Commun. Biol..

[30] Zhong X., Mitchell R., Billstrand C., Thompson E.E., Sakabe N.J., Aneas I., Salamone I.M., Gu J., Sperling A.I., Schoettler N. (2025). Integration of functional genomics and statistical fine-mapping systematically characterizes adult-onset and childhood-onset asthma genetic associations. Genome Med..

[31] Yuan K., Longchamps R.J., Pardinas A.F., Yu M., Chen T.T., Lin S.C., Chen Y., Lam M., Liu R., Xia Y. (2024). Fine-mapping across diverse ancestries drives the discovery of putative causal variants underlying human complex traits and diseases. Nat. Genet..

[32] Kanoni S., Graham S.E., Wang Y., Surakka I., Ramdas S., Zhu X., Clarke S.L., Bhatti K.F., Vedantam S., Winkler T.W. (2022). Implicating genes, pleiotropy, and sexual dimorphism at blood lipid loci through multi-ancestry meta-analysis. Genome Biol..

[33] Hu Y., Haessler J.W., Manansala R., Wiggins K.L., Moscati A., Beiser A., Heard-Costa N.L., Sarnowski C., Raffield L.M., Chung J. (2022). Whole-Genome Sequencing Association Analyses of Stroke and Its Subtypes in Ancestrally Diverse Populations From Trans-Omics for Precision Medicine Project. Stroke.

[34] Cannon M.E., Duan Q., Wu Y., Zeynalzadeh M., Xu Z., Kangas A.J., Soininen P., Ala-Korpela M., Civelek M., Lusis A.J. (2017). Trans-ancestry Fine Mapping and Molecular Assays Identify Regulatory Variants at the ANGPTL8 HDL-C GWAS Locus. G3 Genes Genomes Genet..

[35] Chen M.H., Raffield L.M., Mousas A., Sakaue S., Huffman J.E., Moscati A., Trivedi B., Jiang T., Akbari P., Vuckovic D. (2020). Trans-ethnic and Ancestry-Specific Blood-Cell Genetics in 746,667 Individuals from 5 Global Populations. Cell.

[36] Gay N.R., Gloudemans M., Antonio M.L., Abell N.S., Balliu B., Park Y., Martin A.R., Musharoff S., Rao A.S., Aguet F. (2020). Impact of admixture and ancestry on eQTL analysis and GWAS colocalization in GTEx. Genome Biol..

[37] de Los Campos G., Grueneberg A., Funkhouser S., Perez-Rodriguez P., Samaddar A. (2023). Fine mapping and accurate prediction of complex traits using Bayesian Variable Selection models applied to biobank-size data. Eur. J. Hum. Genet..

[38] Vuckovic D., Bao E.L., Akbari P., Lareau C.A., Mousas A., Jiang T., Chen M.H., Raffield L.M., Tardaguila M., Huffman J.E. (2020). The Polygenic and Monogenic Basis of Blood Traits and Diseases. Cell.

[39] Shao Z., Tang W., Wu H., Kong Y., Hao X. (2025). Incorporating multiple functional annotations to improve polygenic risk prediction accuracy. Cell Genom..

[40] Benner C., Havulinna A.S., Jarvelin M.R., Salomaa V., Ripatti S., Pirinen M. (2017). Prospects of Fine-Mapping Trait-Associated Genomic Regions by Using Summary Statistics from Genome-wide Association Studies. Am. J. Hum. Genet..

[41] Weissbrod O., Hormozdiari F., Benner C., Cui R., Ulirsch J., Gazal S., Schoech A.P., van de Geijn B., Reshef Y., Marquez-Luna C. (2020). Functionally informed fine-mapping and polygenic localization of complex trait heritability. Nat. Genet..

[42] Watanabe K., Taskesen E., van Bochoven A., Posthuma D. (2017). Functional mapping and annotation of genetic associations with FUMA. Nat. Commun..

[43] de Leeuw C.A., Mooij J.M., Heskes T., Posthuma D. (2015). MAGMA: Generalized gene-set analysis of GWAS data. PLoS Comput. Biol..

[44] Wainberg M., Sinnott-Armstrong N., Mancuso N., Barbeira A.N., Knowles D.A., Golan D., Ermel R., Ruusalepp A., Quertermous T., Hao K. (2019). Opportunities and challenges for transcriptome-wide association studies. Nat. Genet..

[45] Consortium G.T. (2020). The GTEx Consortium atlas of genetic regulatory effects across human tissues. Science.

[46] Vosa U., Claringbould A., Westra H.J., Bonder M.J., Deelen P., Zeng B., Kirsten H., Saha A., Kreuzhuber R., Yazar S. (2021). Large-scale cis- and trans-eQTL analyses identify thousands of genetic loci and polygenic scores that regulate blood gene expression. Nat. Genet..

[47] Kerimov N., Tambets R., Hayhurst J.D., Rahu I., Kolberg P., Raudvere U., Kuzmin I., Chowdhary A., Vija A., Teras H.J. (2023). eQTL Catalogue 2023: New datasets, X chromosome QTLs, and improved detection and visualisation of transcript-level QTLs. PLoS Genet..

[48] Giambartolomei C., Vukcevic D., Schadt E.E., Franke L., Hingorani A.D., Wallace C., Plagnol V. (2014). Bayesian test for colocalisation between pairs of genetic association studies using summary statistics. PLoS Genet..

[49] Min J.L., Hemani G., Hannon E., Dekkers K.F., Castillo-Fernandez J., Luijk R., Carnero-Montoro E., Lawson D.J., Burrows K., Suderman M. (2021). Genomic and phenotypic insights from an atlas of genetic effects on DNA methylation. Nat. Genet..

[50] Villicana S., Bell J.T. (2021). Genetic impacts on DNA methylation: Research findings and future perspectives. Genome Biol..

[51] Cao J., Cusanovich D.A., Ramani V., Aghamirzaie D., Pliner H.A., Hill A.J., Daza R.M., McFaline-Figueroa J.L., Packer J.S., Christiansen L. (2018). Joint profiling of chromatin accessibility and gene expression in thousands of single cells. Science.

[52] Sakaue S., Weinand K., Isaac S., Dey K.K., Jagadeesh K., Kanai M., Watts G.F.M., Zhu Z., Brenner M.B., Brenner M.B., Accelerating Medicines Partnership® RA/SLE Program and Network (2024). Tissue-specific enhancer-gene maps from multimodal single-cell data identify causal disease alleles. Nat. Genet..

[53] Su C., Lee D., Jin P., Zhang J. (2025). scMultiMap: Cell-type-specific mapping of enhancers and target genes from single-cell multimodal data. Nat. Commun..

[54] Rao S., Yao Y., Bauer D.E. (2021). Editing GWAS: Experimental approaches to dissect and exploit disease-associated genetic variation. Genome Med..

[55] Gallagher M.D., Chen-Plotkin A.S. (2018). The Post-GWAS Era: From Association to Function. Am. J. Hum. Genet..

[56] Rojano E., Seoane P., Ranea J.A.G., Perkins J.R. (2019). Regulatory variants: From detection to predicting impact. Brief. Bioinform..

[57] Pena-Martinez E.G., Rodriguez-Martinez J.A. (2024). Decoding Non-coding Variants: Recent Approaches to Studying Their Role in Gene Regulation and Human Diseases. Front. Biosci. (Sch. Ed.).

[58] Mumbach M.R., Satpathy A.T., Boyle E.A., Dai C., Gowen B.G., Cho S.W., Nguyen M.L., Rubin A.J., Granja J.M., Kazane K.R. (2017). Enhancer connectome in primary human cells identifies target genes of disease-associated DNA elements. Nat. Genet..

[59] Tambets R., Kolde A., Kolberg P., Love M.I., Alasoo K. (2024). Extensive co-regulation of neighboring genes complicates the use of eQTLs in target gene prioritization. HGG Adv..

[60] Ding R., Wang Q., Gong L., Zhang T., Zou X., Xiong K., Liao Q., Plass M., Li L. (2024). scQTLbase: An integrated human single-cell eQTL database. Nucleic Acids Res..

[61] Gusev A., Ko A., Shi H., Bhatia G., Chung W., Penninx B.W., Jansen R., de Geus E.J., Boomsma D.I., Wright F.A. (2016). Integrative approaches for large-scale transcriptome-wide association studies. Nat. Genet..

[62] Rasooly D., Peloso G.M., Giambartolomei C. (2022). Bayesian Genetic Colocalization Test of Two Traits Using coloc. Curr. Protoc..

[63] Wu Y., Broadaway K.A., Raulerson C.K., Scott L.J., Pan C., Ko A., He A., Tilford C., Fuchsberger C., Locke A.E. (2019). Colocalization of GWAS and eQTL signals at loci with multiple signals identifies additional candidate genes for body fat distribution. Hum. Mol. Genet..

[64] Zhang Y., Wang M., Li Z., Yang X., Li K., Xie A., Dong F., Wang S., Yan J., Liu J. (2024). An overview of detecting gene-trait associations by integrating GWAS summary statistics and eQTLs. Sci. China Life Sci..

[65] Li B., Ritchie M.D. (2021). From GWAS to Gene: Transcriptome-Wide Association Studies and Other Methods to Functionally Understand GWAS Discoveries. Front. Genet..

[66] Evans P., Nagai T., Konkashbaev A., Zhou D., Knapik E.W., Gamazon E.R. (2024). Transcriptome-Wide Association Studies (TWAS): Methodologies, Applications, and Challenges. Curr. Protoc..

[67] Gaulton K.J., Preissl S., Ren B. (2023). Interpreting non-coding disease-associated human variants using single-cell epigenomics. Nat. Rev. Genet..

[68] Zhang K., Hocker J.D., Miller M., Hou X., Chiou J., Poirion O.B., Qiu Y., Li Y.E., Gaulton K.J., Wang A. (2021). A single-cell atlas of chromatin accessibility in the human genome. Cell.

[69] Chiou J., Geusz R.J., Okino M.L., Han J.Y., Miller M., Melton R., Beebe E., Benaglio P., Huang S., Korgaonkar K. (2021). Interpreting type 1 diabetes risk with genetics and single-cell epigenomics. Nature.

[70] Pratt B.M., Won H. (2022). Advances in profiling chromatin architecture shed light on the regulatory dynamics underlying brain disorders. Semin. Cell Dev. Biol..

[71] Kiessling P., Kuppe C. (2024). Spatial multi-omics: Novel tools to study the complexity of cardiovascular diseases. Genome Med..

[72] Jia P., Zhao Z. (2014). Network.assisted analysis to prioritize GWAS results: Principles, methods and perspectives. Hum. Genet..

[73] Ochoa D., Hercules A., Carmona M., Suveges D., Gonzalez-Uriarte A., Malangone C., Miranda A., Fumis L., Carvalho-Silva D., Spitzer M. (2021). Open Targets Platform: Supporting systematic drug-target identification and prioritisation. Nucleic Acids Res..

[74] Yu G., Tam C.H.T., Lim C.K.P., Shi M., Lau E.S.H., Ozaki R., Lee H.M., Ng A.C.W., Hou Y., Fan B. (2025). Type 2 diabetes pathway-specific polygenic risk scores elucidate heterogeneity in clinical presentation, disease progression and diabetic complications in 18,217 Chinese individuals with type 2 diabetes. Diabetologia.

[75] Kyono Y., Kitzman J.O., Parker S.C.J. (2019). Genomic annotation of disease-associated variants reveals shared functional contexts. Diabetologia.

[76] Hsieh K., Wang Y., Chen L., Zhao Z., Savitz S., Jiang X., Tang J., Kim Y. (2021). Drug repurposing for COVID-19 using graph neural network and harmonizing multiple evidence. Sci. Rep..

[77] McDonagh E.M., Trynka G., McCarthy M., Holzinger E.R., Khader S., Nakic N., Hu X., Cornu H., Dunham I., Hulcoop D. (2024). Human Genetics and Genomics for Drug Target Identification and Prioritization: Open Targets’ Perspective. Annu. Rev. Biomed. Data Sci..

[78] Karim M.R., Islam T., Shajalal M., Beyan O., Lange C., Cochez M., Rebholz-Schuhmann D., Decker S. (2023). Explainable AI for Bioinformatics: Methods, Tools and Applications. Brief. Bioinform..

[79] Mann M., Kumar C., Zeng W.F., Strauss M.T. (2021). Artificial intelligence for proteomics and biomarker discovery. Cell Syst..

[80] Watson D.S. (2022). Interpretable machine learning for genomics. Hum. Genet..

[81] Novakovsky G., Fornes O., Saraswat M., Mostafavi S., Wasserman W.W. (2023). ExplaiNN: Interpretable and transparent neural networks for genomics. Genome Biol..

[82] Majdandzic A., Rajesh C., Tang A., Toneyan S., Labelson E., Tripathy R., Koo P.K. (2022). Selecting deep neural networks that yield consistent attribution-based interpretations for genomics. Proc. Mach. Learn. Res..

[83] Chen V., Yang M., Cui W., Kim J.S., Talwalkar A., Ma J. (2024). Applying interpretable machine learning in computational biology-pitfalls, recommendations and opportunities for new developments. Nat. Methods.

[84] Yang K., Cheng J., Cao S., Pan X., Shen H.B., Cheng J., Yuan Y. (2025). Integration of multi-source gene interaction networks and omics data with graph attention networks to identify novel disease genes. Bioinformatics.

[85] Jia X., Luo W., Li J., Xing J., Sun H., Wu S., Su X. (2024). A deep learning framework for predicting disease-gene associations with functional modules and graph augmentation. BMC Bioinform..

[86] Abe S., Tago S., Yokoyama K., Ogawa M., Takei T., Imoto S., Fuji M. (2023). Explainable AI for Estimating Pathogenicity of Genetic Variants Using Large-Scale Knowledge Graphs. Cancers.

[87] Azodi C.B., Tang J., Shiu S.H. (2020). Opening the Black Box: Interpretable Machine Learning for Geneticists. Trends Genet..

[88] Roman T.S., Mohlke K.L. (2018). Functional genomics and assays of regulatory activity detect mechanisms at loci for lipid traits and coronary artery disease. Curr. Opin. Genet. Dev..

[89] Hukerikar N., Hingorani A.D., Asselbergs F.W., Finan C., Schmidt A.F. (2024). Prioritising genetic findings for drug target identification and validation. Atherosclerosis.

[90] Ang M.Y., Chen L., Song L., Lipovich L., Choo S.W. (2026). Responsible Use of Large Language Models in Microbial Genomics and Bioinformatics: A Life-Science Framework for Reliability, Reproducibility, and Risk-Aware Interpretation. Life.

[91] Holmes M.V., Richardson T.G., Ference B.A., Davies N.M., Davey Smith G. (2021). Integrating genomics with biomarkers and therapeutic targets to invigorate cardiovascular drug development. Nat. Rev. Cardiol..

[92] Nelson M.R., Tipney H., Painter J.L., Shen J., Nicoletti P., Shen Y., Floratos A., Sham P.C., Li M.J., Wang J. (2015). The support of human genetic evidence for approved drug indications. Nat. Genet..

[93] Zhou J., Troyanskaya O.G. (2015). Predicting effects of noncoding variants with deep learning-based sequence model. Nat. Methods.

[94] Avsec Z., Agarwal V., Visentin D., Ledsam J.R., Grabska-Barwinska A., Taylor K.R., Assael Y., Jumper J., Kohli P., Kelley D.R. (2021). Effective gene expression prediction from sequence by integrating long-range interactions. Nat. Methods.

[95] Daghlas I., Gill D. (2023). Mendelian randomization as a tool to inform drug development using human genetics. Camb. Prism. Precis. Med..

[96] Zheng J., Haberland V., Baird D., Walker V., Haycock P.C., Hurle M.R., Gutteridge A., Erola P., Liu Y., Luo S. (2020). Phenome-wide Mendelian randomization mapping the influence of the plasma proteome on complex diseases. Nat. Genet..

[97] Henry A., Gordillo-Maranon M., Finan C., Schmidt A.F., Ferreira J.P., Karra R., Sundstrom J., Lind L., Arnlov J., Zannad F. (2022). Therapeutic Targets for Heart Failure Identified Using Proteomics and Mendelian Randomization. Circulation.

[98] Xu D., Lu J., Yang Y., Hu W., Chen J., Xue J., Yang S., Cao N., Hu H., Qian N. (2025). Identifying novel drug targets for calcific aortic valve disease through Mendelian randomization. Atherosclerosis.

[99] Ning Z., Huang Y., Lu H., Zhou Y., Tu T., Ouyang F., Liu Y., Liu Q. (2024). Novel Drug Targets for Atrial Fibrillation Identified Through Mendelian Randomization Analysis of the Blood Proteome. Cardiovasc. Drugs Ther..

[100] Sun J., Zhao J., Jiang F., Wang L., Xiao Q., Han F., Chen J., Yuan S., Wei J., Larsson S.C. (2023). Identification of novel protein biomarkers and drug targets for colorectal cancer by integrating human plasma proteome with genome. Genome Med..

[101] Interleukin-6 Receptor Mendelian Randomisation Analysis C., Swerdlow D.I., Holmes M.V., Kuchenbaecker K.B., Engmann J.E., Shah T., Sofat R., Guo Y., Chung C., Peasey A. (2012). The interleukin-6 receptor as a target for prevention of coronary heart disease: A mendelian randomisation analysis. Lancet.

[102] Bai Y., Wang J., Feng X., Xie L., Qin S., Ma G., Zhang F. (2024). Identification of drug targets for Sjogren’s syndrome: Multi-omics Mendelian randomization and colocalization analyses. Front. Immunol..

[103] Przybyla L., Gilbert L.A. (2022). A new era in functional genomics screens. Nat. Rev. Genet..

[104] Shi H., Doench J.G., Chi H. (2023). CRISPR screens for functional interrogation of immunity. Nat. Rev. Immunol..

[105] Inoue F., Ahituv N. (2015). Decoding enhancers using massively parallel reporter assays. Genomics.

[106] McAfee J.C., Bell J.L., Krupa O., Matoba N., Stein J.L., Won H. (2022). Focus on your locus with a massively parallel reporter assay. J. Neurodev. Disord..

[107] Sonti S., Littleton S.H., Pahl M.C., Zimmerman A.J., Chesi A., Palermo J., Lasconi C., Brown E.B., Pippin J.A., Wells A.D. (2024). Perturbation of the insomnia WDR90 genome-wide association studies locus pinpoints rs3752495 as a causal variant influencing distal expression of neighboring gene, PIG-Q. Sleep.

[108] Lee S., McAfee J.C., Lee J., Gomez A., Ledford A.T., Clarke D., Min H., Gerstein M.B., Boyle A.P., Sullivan P.F. (2025). Massively parallel reporter assay investigates shared genetic variants of eight psychiatric disorders. Cell.

[109] King E.A., Davis J.W., Degner J.F. (2019). Are drug targets with genetic support twice as likely to be approved? Revised estimates of the impact of genetic support for drug mechanisms on the probability of drug approval. PLoS Genet..

[110] Sun B.B., Maranville J.C., Peters J.E., Stacey D., Staley J.R., Blackshaw J., Burgess S., Jiang T., Paige E., Surendran P. (2018). Genomic atlas of the human plasma proteome. Nature.

[111] Ferkingstad E., Sulem P., Atlason B.A., Sveinbjornsson G., Magnusson M.I., Styrmisdottir E.L., Gunnarsdottir K., Helgason A., Oddsson A., Halldorsson B.V. (2021). Large-scale integration of the plasma proteome with genetics and disease. Nat. Genet..

[112] Gill D., Georgakis M.K., Walker V.M., Schmidt A.F., Gkatzionis A., Freitag D.F., Finan C., Hingorani A.D., Howson J.M.M., Burgess S. (2021). Mendelian randomization for studying the effects of perturbing drug targets. Wellcome Open Res..

[113] Schmidt A.F., Finan C., Gordillo-Maranon M., Asselbergs F.W., Freitag D.F., Patel R.S., Tyl B., Chopade S., Faraway R., Zwierzyna M. (2020). Genetic drug target validation using Mendelian randomisation. Nat. Commun..

[114] Gupta C., Xu J., Jin T., Khullar S., Liu X., Alatkar S., Cheng F., Wang D. (2022). Single-cell network biology characterizes cell type gene regulation for drug repurposing and phenotype prediction in Alzheimer’s disease. PLoS Comput. Biol..

[115] Jagadeesh K.A., Dey K.K., Montoro D.T., Mohan R., Gazal S., Engreitz J.M., Xavier R.J., Price A.L., Regev A. (2022). Identifying disease-critical cell types and cellular processes by integrating single-cell RNA-sequencing and human genetics. Nat. Genet..

[116] Zhang M.J., Hou K., Dey K.K., Sakaue S., Jagadeesh K.A., Weinand K., Taychameekiatchai A., Rao P., Pisco A.O., Zou J. (2022). Polygenic enrichment distinguishes disease associations of individual cells in single-cell RNA-seq data. Nat. Genet..

[117] Koscielny G., An P., Carvalho-Silva D., Cham J.A., Fumis L., Gasparyan R., Hasan S., Karamanis N., Maguire M., Papa E. (2017). Open Targets: A platform for therapeutic target identification and validation. Nucleic Acids Res..

[118] Finan C., Gaulton A., Kruger F.A., Lumbers R.T., Shah T., Engmann J., Galver L., Kelley R., Karlsson A., Santos R. (2017). The druggable genome and support for target identification and validation in drug development. Sci. Transl. Med..

[119] Walker V.M., Davey Smith G., Davies N.M., Martin R.M. (2017). Mendelian randomization: A novel approach for the prediction of adverse drug events and drug repurposing opportunities. Int. J. Epidemiol..

[120] Loos R.J.F., Yeo G.S.H. (2022). The genetics of obesity: From discovery to biology. Nat. Rev. Genet..

[121] Pulit S.L., Stoneman C., Morris A.P., Wood A.R., Glastonbury C.A., Tyrrell J., Yengo L., Ferreira T., Marouli E., Ji Y. (2019). Meta-analysis of genome-wide association studies for body fat distribution in 694 649 individuals of European ancestry. Hum. Mol. Genet..

[122] Shungin D., Winkler T.W., Croteau-Chonka D.C., Ferreira T., Locke A.E., Magi R., Strawbridge R.J., Pers T.H., Fischer K., Justice A.E. (2015). New genetic loci link adipose and insulin biology to body fat distribution. Nature.

[123] Mahajan A., Spracklen C.N., Zhang W., Ng M.C.Y., Petty L.E., Kitajima H., Yu G.Z., Rueger S., Speidel L., Kim Y.J. (2022). Multi-ancestry genetic study of type 2 diabetes highlights the power of diverse populations for discovery and translation. Nat. Genet..

[124] Banerjee D., Girirajan S. (2025). Discovery of obesity genes through cross-ancestry analysis. Nat. Commun..

[125] Ang M.Y., Takeuchi F., Kato N. (2023). Deciphering the genetic landscape of obesity: A data-driven approach to identifying plausible causal genes and therapeutic targets. J. Hum. Genet..

[126] Broadaway K.A., Brotman S.M., Rosen J.D., Currin K.W., Alkhawaja A.A., Etheridge A.S., Wright F., Gallins P., Jima D., Zhou Y.H. (2024). Liver eQTL meta-analysis illuminates potential molecular mechanisms of cardiometabolic traits. Am. J. Hum. Genet..

[127] van Duijvenboden S., Ramirez J., Young W.J., Olczak K.J., Ahmed F., Alhammadi M., Bell C.G., Morris A.P., Munroe P.B., International Consortium of Blood Pressure (2023). Integration of genetic fine-mapping and multi-omics data reveals candidate effector genes for hypertension. Am. J. Hum. Genet..

[128] Ray A., Alabarse P., Malik R., Sargurupremraj M., Bernhagen J., Dichgans M., Baumeister S.E., Georgakis M.K. (2025). Single-cell transcriptome-wide Mendelian randomization and colocalization analyses uncover cell-specific mechanisms in atherosclerotic cardiovascular disease. Am. J. Hum. Genet..

[129] Aragam K.G., Jiang T., Goel A., Kanoni S., Wolford B.N., Atri D.S., Weeks E.M., Wang M., Hindy G., Zhou W. (2022). Discovery and systematic characterization of risk variants and genes for coronary artery disease in over a million participants. Nat. Genet..

[130] Zheng S., Tsao P.S., Pan C. (2024). Abdominal aortic aneurysm and cardiometabolic traits share strong genetic susceptibility to lipid metabolism and inflammation. Nat. Commun..

[131] Wang S., Liu S., Zhang H., Sun L., Tan H., Shi Y., Pan L., Geng M., Chen M., Gao B. (2026). Integrative functional genomics and fine-mapping identify regulatory mechanisms of multivariate obesity GWAS and its cardiometabolic implications. Metabolism.

[132] Cheng C., Liu Y., Sun L., Fan J., Sun X., Zheng J.S., Zheng L., Zhu Y., Zhou D. (2025). Integrative metabolomics and genomics reveal molecular signatures for type 2 diabetes and its cardiovascular complications. Cardiovasc. Diabetol..

[133] Chami N., Wang Z., Svenstrup V., Obrero V.D., Hemerich D., Huang Y., Dashti H., Manitta E., Preuss M.H., North K.E. (2025). Genetic subtyping of obesity reveals biological insights into the uncoupling of adiposity from its cardiometabolic comorbidities. Nat. Med..

[134] Ye C., Dou C., Liu D., Kong L., Chen M., Xu M., Xu Y., Li M., Zhao Z., Zheng J. (2025). Multivariate genome-wide analyses of insulin resistance unravel novel loci and therapeutic targets for cardiometabolic health. Nat. Commun..

[135] Xiong R., Aiken E., Caldwell R., Vernon S.D., Kozhaya L., Gunter C., Bateman L., Unutmaz D., Oh J. (2025). AI-driven multi-omics modeling of myalgic encephalomyelitis/chronic fatigue syndrome. Nat. Med..

[136] Wang T., He Q., Chan K.H.K. (2025). A Multi-omics approach to identify and validate shared genetic architecture in rheumatoid arthritis, multiple sclerosis, and type 1 diabetes: Integrating GWAS, GEO, MSigDB, and scRNA-seq data. Funct. Integr. Genom..

[137] Claussnitzer M., Dankel S.N., Kim K.H., Quon G., Meuleman W., Haugen C., Glunk V., Sousa I.S., Beaudry J.L., Puviindran V. (2015). FTO Obesity Variant Circuitry and Adipocyte Browning in Humans. N. Engl. J. Med..

[138] Loos R.J., Lindgren C.M., Li S., Wheeler E., Zhao J.H., Prokopenko I., Inouye M., Freathy R.M., Attwood A.P., Beckmann J.S. (2008). Common variants near MC4R are associated with fat mass, weight and risk of obesity. Nat. Genet..

[139] Musunuru K., Strong A., Frank-Kamenetsky M., Lee N.E., Ahfeldt T., Sachs K.V., Li X., Li H., Kuperwasser N., Ruda V.M. (2010). From noncoding variant to phenotype via SORT1 at the 1p13 cholesterol locus. Nature.

[140] Cohen J.C., Boerwinkle E., Mosley T.H., Hobbs H.H. (2006). Sequence variations in PCSK9, low LDL, and protection against coronary heart disease. N. Engl. J. Med..

[141] Bennet A.M., Di Angelantonio E., Ye Z., Wensley F., Dahlin A., Ahlbom A., Keavney B., Collins R., Wiman B., de Faire U. (2007). Association of apolipoprotein E genotypes with lipid levels and coronary risk. JAMA.

[142] Whalen S., Schreiber J., Noble W.S., Pollard K.S. (2022). Navigating the pitfalls of applying machine learning in genomics. Nat. Rev. Genet..

[143] Udler M.S., Kim J., von Grotthuss M., Bonas-Guarch S., Cole J.B., Chiou J., Christopher D., Boehnke M., Laakso M., Atzmon G. (2018). Type 2 diabetes genetic loci informed by multi-trait associations point to disease mechanisms and subtypes: A soft clustering analysis. PLoS Med..

[144] Suzuki K., Hatzikotoulas K., Southam L., Taylor H.J., Yin X., Lorenz K.M., Mandla R., Huerta-Chagoya A., Melloni G.E.M., Kanoni S. (2024). Genetic drivers of heterogeneity in type 2 diabetes pathophysiology. Nature.

[145] Manning A.K., Hivert M.F., Scott R.A., Grimsby J.L., Bouatia-Naji N., Chen H., Rybin D., Liu C.T., Bielak L.F., Prokopenko I. (2012). A genome-wide approach accounting for body mass index identifies genetic variants influencing fasting glycemic traits and insulin resistance. Nat. Genet..

[146] Brown A.E., Walker M. (2016). Genetics of Insulin Resistance and the Metabolic Syndrome. Curr. Cardiol. Rep..

[147] Gordillo-Maranon M., Zwierzyna M., Charoen P., Drenos F., Chopade S., Shah T., Engmann J., Chaturvedi N., Papacosta O., Wannamethee G. (2021). Validation of lipid-related therapeutic targets for coronary heart disease prevention using human genetics. Nat. Commun..

[148] Schnitzler G.R., Kang H., Fang S., Angom R.S., Lee-Kim V.S., Ma X.R., Zhou R., Zeng T., Guo K., Taylor M.S. (2024). Convergence of coronary artery disease genes onto endothelial cell programs. Nature.

[149] Wunnemann F., Fotsing Tadjo T., Beaudoin M., Lalonde S., Lo K.S., Kleinstiver B.P., Lettre G. (2023). Multimodal CRISPR perturbations of GWAS loci associated with coronary artery disease in vascular endothelial cells. PLoS Genet..

[150] Zekavat S.M., Raghu V.K., Trinder M., Ye Y., Koyama S., Honigberg M.C., Yu Z., Pampana A., Urbut S., Haidermota S. (2022). Deep Learning of the Retina Enables Phenome- and Genome-Wide Analyses of the Microvasculature. Circulation.

[151] Xie Z., Zhang T., Kim S., Lu J., Zhang W., Lin C.H., Wu M.R., Davis A., Channa R., Giancardo L. (2024). iGWAS: Image-based genome-wide association of self-supervised deep phenotyping of retina fundus images. PLoS Genet..

[152] Schaid D.J., Chen W., Larson N.B. (2018). From genome-wide associations to candidate causal variants by statistical fine-mapping. Nat. Rev. Genet..

[153] Rufibach K. (2010). Use of Brier score to assess binary predictions. J. Clin. Epidemiol..

[154] Van Calster B., McLernon D.J., van Smeden M., Wynants L., Steyerberg E.W., On behalf of Topic Group ‘Evaluating Diagnostic Tests and Prediction Models’ of the STRATOS Initiative (2019). Calibration: The Achilles heel of predictive analytics. BMC Med..

[155] Vickers A.J., Elkin E.B. (2006). Decision curve analysis: A novel method for evaluating prediction models. Med. Decis. Mak..

[156] Alexander D.H., Lange K. (2011). Stability selection for genome-wide association. Genet. Epidemiol..

